# Au-Decorated Dragonfly Wing Bioscaffold Arrays as Flexible Surface-Enhanced Raman Scattering (SERS) Substrate for Simultaneous Determination of Pesticide Residues

**DOI:** 10.3390/nano8050289

**Published:** 2018-04-28

**Authors:** Mingli Wang, Guochao Shi, Yanying Zhu, Yuhong Wang, Wanli Ma

**Affiliations:** 1 Key Laboratory for Microstructural Material Physics of Hebei Province, School of Science, Yanshan University, Qinhuangdao, Hebei 066004, China; sgc@stumail.ysu.edu.cn (G.S.); wangyh@stumail.ysu.edu.cn (Y.W.); 2 Department of Mathematics, NC State University, Raleigh, NC 276968205, USA; wanlimaphil@gmail.com (W.M.)

**Keywords:** SERS, dragonfly wing, magnetron sputtering, pesticide residue

## Abstract

Rapid sampling and multicomponent analysis are vital in pesticide residue detection. In this work, we proposed a SERS platform to detect three kinds of pesticides on apple peels simultaneously by a straightforward “press and peel off” method. The flexible Au/dragonfly wing (Au/DW) substrate was obtained from sputtering Au nanoislands on DW bioscaffold arrays by a simple direct current (DC) magnetron sputtering system. The high-performance substrate exhibited a low limit of detection (LOD) to 4-aminothiophenol (4-ATP) (10^−9^ M), outstanding reproducibility (less than 12.15%), good stability and suitability in multifold pesticide residues detection. Considering its excellent sample collection efficiency, the Au/DW substrate was employed to solve critical pesticide residue problems for detection of acephate (APT), cypermethrin (CPT), tsumacide (MTMC) and their multiple components on apple peels. The results show that the LOD was 10^−3^ ng/cm^2^ for APT obtained on the apple surface with a calculation equation of y = 0.26x + 6.68 and a determination coefficient (R^2^) of 0.970. Additionally, the LOD values for CPT and MTMC were 10^−3^ ng/cm^2^ and 10^−4^ ng/cm^2^, respectively. The finding in this work may provide a promising biomimetic SERS platform for on-spot detection of other organic pollutants in the food industry and inenvironmental protection.

## 1. Introduction

In recent years, with the rapid development of economy, consumers and organizations are increasingly concerned about food safety issues which have developed into a worldwide problem [1]. On the other hand, many detection techniques such as high-performance liquid chromatography (HPLC) [2], colorimetry [3], gas chromatography mass spectroscopy (GC-MS) [4], fluorescence analysis [5], and enzyme inhibition [6] have been widely applied to detect pesticide residues on vegetables and fruits for their accurate measurement. However, these typical methods are usually time-consuming, expensive, laborious, or require complicated pretreatments which cannot fully meet the need for the convenient, rapid, and cost-effective detection in the modern food industry. Therefore, the development of simpler and more efficient detection methods involving minimum cost and minimal sample pretreatment is a challenge, particularly when it is required for on-site analysis.

The pesticide residues in fruit, vegetables and crops have drawn wide attention due to the unscientific abuse of pesticides, therefore, there has been a powerful driving force to seek techniques for the rapid detection of pesticide residues [7]. Among all the reported techniques, surface-enhanced Raman scattering (SERS) has been proven to be a powerful fingerprinting technique, allowing for simple, rapid, highly sensitive, and non-destructive identification of trace molecules [8,9,10]. By integrating all these advantages, SERS technique can partly remedy the disadvantages of other approaches and has been one of the best candidates applied in public security [11], biomedical science [12], environmental monitoring [13] and food analysis [14]. It is widely accepted that the amplification of the Raman signals can be confirmed as the result of two comprehensive effects: electromagnetic enhancement (EM) and chemical enhancement (CM) mechanism. Due to the unique localized surface plasmon resonance (LSPR) and the local electromagnetic field enhancement effect at appropriate nanostructural junctions (“hot spots”) between metallic nanostructures, EM mechanism is mainly contributed to the SERS enhancement [15,16]. Because SERS technique can provide “fingerprint-like” spectrum information, noble metal nanomaterial SERS substrates based on LSPR-excited localized field enhancement have been widely applied to the detection of multifold pesticide residues. Chen et al. reported a kind of novel strategy based on Au nanoparticles (NPs)/tape as flexible and adhesive SERS substrate. The high-performance SERS substrate was employed to detect parathion-methyl in real samples and the LOD was as low as 2.60 ng/cm^2^ [17]. Recently, a flexible and transparent Ag-nanocubes@polyethylene (PE) film as a cut-and-paste SERS substrate was put forward to rapid detecting of methyl parathion on oranges and the LOD was located at approximately 10 nM [18]. Zhu et al. have developed a simple method to fabricate “dynamic SERS” (D-SERS) based on depositing silver nanoparticles on the surface of filter paper. SERS experiments were performed to detect thiram and paraoxon residues at various peels [19]. Nanostar-shaped Au NPs were successfully formed seed-growth method. This interesting SERS substrate was applied to detect thiram on apple peels which satisfied the demand for actual applications [20]. Furthermore, rapid simultaneous detection of multi-pesticide residues in food matrices can be achieved through SERS technique because of the narrow Raman bands with minimal overlapping, which is important for human health and food safety [21]. Inspired by gecko, Wang et al. developed a gecko-inspired nanotentacle SERS platform which was used to accurately detect and quantify three pesticide mixtures (methyl parathion, thiram and malachite green) on cucumber [22].

**Figure 1:**
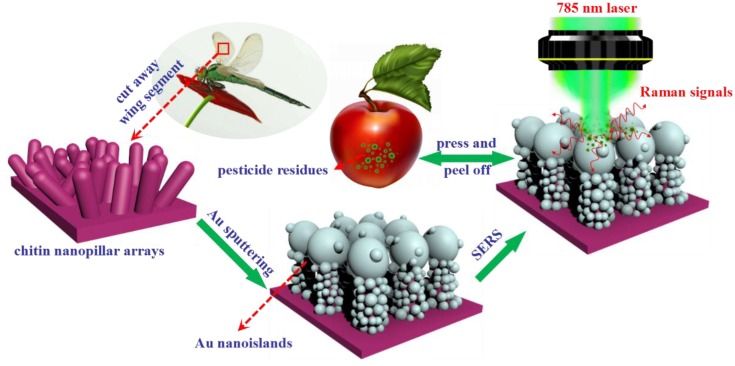
Schematic illustration of the fabrication process of the SERS substrate through sputtering Au nanoislands on dragonfly wing and SERS measurement of Au/DW substrate by Raman system.

Here, we report a facile approach to fabricate three-dimensional (3D) biomimetic array as flexible SERS substrate with high-density “hot spots”, excellent stability and reproducibility. Compared to the traditional rigid substrates and those the substrates mentioned above, our irregular Au/DW substrates are more flexible, eco-friendly, stable, and sensitive, which can be efficiently used in practical on-site multipesticide detection. The fabrication process for 3D Au/dragonfly wing (Au/DW) was illustrated in Figure 1. In brief, after cleansed thoroughly, the DWs were segmented into a series of small sections. Then, the Au nanoislands were decorated onto the biological scaffolds by DC magnetron sputtering system. It should be highlighted that this simple physical sputtering technique strategy effectively avoided unnecessary, expensive, and complicated stages in chemical modification methods which link the metal NPs onto the scaffolds [23]. Subsequently, the fabricated Au/DW substrate which was prepared with the sputtering time of 45 min was employed in the label-free detection and differentiation of the pesticide residues including acephate (APT), cypermethrin (CPT), tsumacide (MTMC) from actual sample surfaces via a simple “press and peel off” approach. The results indicated that through greatly enhanced Raman signals obtained by Au nanoislands decorated DW SERS substrates, the pesticide residues of APT, CPT and MTMC were effectively detected on apple peels with the limit of detection (LOD) as low as 10^−3^ ng/cm^2^, 10^−3^ ng/cm^2^ and 10^−4^ ng/cm^2^. The flexible and high-performance SERS substrates clearly demonstrate its potential as rapid, non-destructive, and sensitive SERS detecting platform in trace detection and biosensing applications.

## 2. Experimental

### 2.1. Materials and Instruments

Acetone and ethanol were purchased from Aladdin, Shanghai, China. Acephate, cypermethrin, 4-aminothiophenol and tsumacide were obtained from J&K Scientific Ltd. (Beijing, China). The sputtering target of gold (99.99%) was purchased from ZhongNuo Advanced Material (Beijing, China) Technology Co., Ltd. Dragonfly wings were supplied by Hebei University of Environmental Engineering. Experiments with dragonfly wing complied with the accepted ethical standards and were approved by the Ethical Review Board of Yanshan University on 15 January 2018. Other reagents used in the experiments, unless mentioned otherwise, were of analytical grade and used without further purification. Deionized water (>18.00 MΩ) was used for all solution preparations.

### 2.2. Sample Preparation

The Au/DW substrates were fabricated by a simple and eco-friendly method. Firstly, prior to the preparation of Au/DW substrates, a series of dragonfly wings with an area of 1 × 1 cm^2^ were cleaned by acetone, ethanol, and deionized water for 20 min in turn to remove the residual impurities, followed by natural drying. In general, an integrated dragonfly wing can be made into 4 SERS substrates. Secondly, the Au nanoislands were deposited on the DW surface by the high vacuum direct current (DC) magnetron sputtering system (LAB 18). Before the system set to work, pumping the pressure in the vacuum chamber to 8 × 10^−4^ Pa with a vacuum pump. Then, high purity Ar gas was filled into the vacuum chamber to keep the pressure at 6 × 10^−1^ Pa with a flow rate of 10 mL/min. The circular sputtering target gold (99.99%) with a diameter of 50.8 mm and a thickness of 3.175 mm was used in the experiments. The power supply was 70 W which was operated at a crystal-controlled frequency of 13.56 MHz. During the sputtering process, the sputtering voltage was controlled to be 90 V, the current was 170 mA, the frequency was 13.56 MHz and the sputtering rate was about 0.03 nm/s. All the sputtering deposition processes were performed at room temperature. The contrast analysis indicated that 45 min deposited time of Au nanoislands has the best SERS sensitivity. Therefore, the Au/DW with a deposited time of 45 min was used in all tests.

### 2.3. Characterization

Field emission scanning electron microscopy (FE-SEM) (JEOL JSM-2100) (Hitachi Ltd., Tokyo, Japan) system was performed to investigate the morphological features and microstructures of DW and Au/DW at room temperature. The Raman spectra were recorded by the confocal microscope Raman spectrometer system (inVia) using a 785 nm laser excitation light source with 0.5 mW power. A 50×, 0.75 NA objective lens with an appropriate working distance focused the excitation beam onto samples with a spot size of ca 1 μm and the spectral resolution was 1 cm^−1^. The exposure time for each SERS measurement was typically set to be 10 s. Unless specially instructed, the accumulation time and the laser power were the same for all Raman spectra. For the detection of 4-ATP, a 1 × 1 cm^2^ of the Au/DW substrate was immersed into the different concentrations (10^−3^ M–10^−9^ M) of 4-ATP ethanol solution and standing for 1 h to reach the adsorption equilibrium. Afterwards, the samples with 4-ATP were dried under ambient conditions for 5 min. All the data were averaged over 10 randomly selected positions. During the SERS mapping setup, in the selected area, the step size was set to be 1 μm, and the accumulative time was 3 s. The information of regional mapping was obtained by spot scanning. The color depth of each grid represents the size of the Raman intensity of the scanning position.

### 2.4. Detection of Pesticide Residues on Apple Peels

First, deionized water and ethanol solution were used to clean the residual contaminants on the apple’s surface. Then, a clean fruit knife was used to cut the apple peels into ~1 cm^2^ pieces. Subsequently, 10 μL of the prepared pesticide solutions with different concentrations were directly sprayed onto the surface of the apple peels. After natural drying at room temperature, 10 μL ethanol solution was added to the pretreated apple peel samples. Last, the Au/DW substrate was pressed to the samples until completely dry and then peeled off for further analysis. The detection of other pesticide residues mentioned in this work also followed the above steps. To ensure that the Raman signals of pesticide molecules were successfully obtained, the SERS measurements were performed 5 times randomly within the treated apple peels and the collected SERS data were averaged.

## 3. Results and Discussion

### 3.1. Morphology Characterization

**Figure 2:**
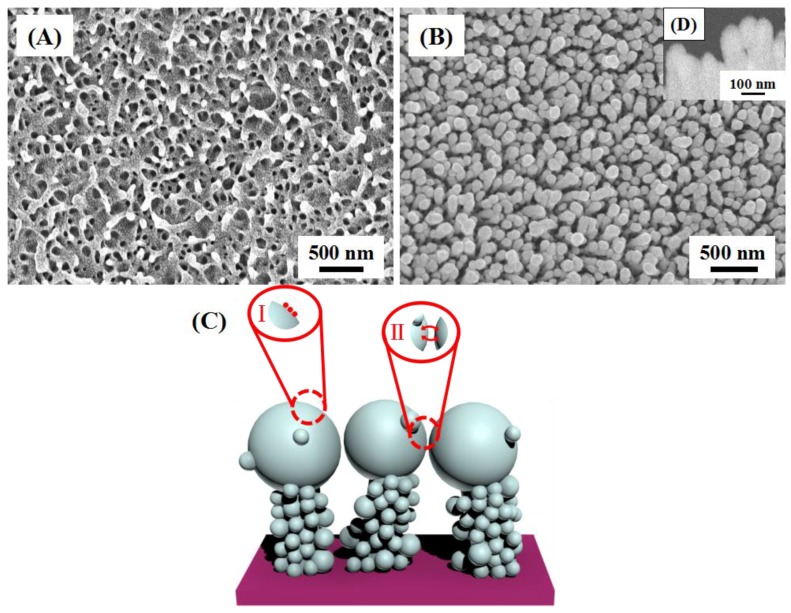
FE-SEM images of (**A**) DW from the top view and (**B**) Au/DW substrates obtained by sputtering time of 45 min. (**C**) Simulated distribution of different types of “hot spots” on Au/DW substrate. (**D**) side-view SEM image of Au/DW.

Figure 2A shows the surface morphology of DW. Obviously, it exhibited large-scale pillar nanostructures which processed a high degree irregularity could provide a high density of holders for decorating Au nanoislands in different dimensions. Surface-view micrographs revealed that the epicuticular layers were composed of multi-column nanopillars with the average height of 200 nm. The space between the nearest-neighbor nanopillars was approximately 180 ± 30 nm and the average diameter of the round tops was 80 ± 20 nm, although somewhat randomly distributed [24]. Figure 2B of the Au nanoislands decorated DW clearly shows that many Au nanoislands have formed within 1 cm^2^ without damaging the original microstructure of the DW, resulting in a complex irregular nanocomposite array. When the sputtering time was set to be 45 min, the nanopillar structures disappeared, while the Au nanoislands with the average value of 100 ± 5 nm in diameter were formed on the tops. Meanwhile, the Au nanoislands were also extended along the vertical sides of the nanopillars, but the size was smaller than those located on the tops. This image was a representative one taken at different regions of the Au/DW substrates. Clearly, the distribution of the Au nanoislands is more homogeneous on the tops of the nanopillars and there were plentiful suitable nanogaps which could offer a great deal of “hot spots” and further enhanced the scattering cross-section. The distribution of two types of “hot spots” were displayed on the surface of the whole substrate in a simulated way as exhibited in Figure 2C. Type “I” was formed on a single Au nanoisland where the LSPR effect was excited under the laser. Type “II” was generated between the neighboring Au nanoislands on the top of the nanopillars. Due to the irregular distribution of the nanopillars on the surface of the DW, the whole substrate had more opportunities to form “hot spots”, thus, enhancing the near-field enhancement effects under incident light [25]. Figure 2D shows the side-view SEM of Au/DW substrate. Obviously, the average diameter of Au nanoislands was about 100 nm on the top and the side of the protrusions were also fully covered with small nanoislands. As can we see from the side-view SEM of Au/DW substrate, the nanogaps between two adjacent Au nanoislands on the top of the nanopillars were all less than 10 nm, which indicated that when the nanogaps between the noble metal nanostructure were small, effective coupling of LSPR would be caused. Thus, the higher intensity of the Raman band of the analyte corresponded to the presence of the Au nanoislands that were in the top of the nanopillars.

### 3.2. SERS Performances of the Au/DW Substrate

**Figure 3:**
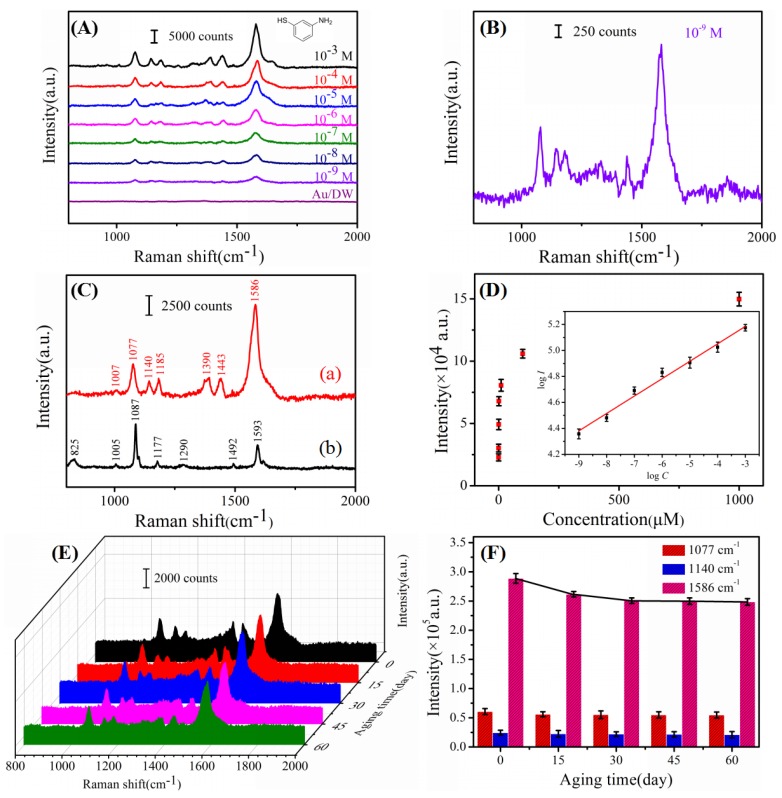
(**A**) The Raman spectra of 4-ATP solution at different concentrations detected by the Au/DW substrate and Raman spectrum of Au/DW substrate; (**B**) The Raman spectra of 10^−9^ M 4-ATP; (**C**) SERS spectrum of 10^−3^ M 4-ATP on the Au/DW substrate (**a**) and Raman spectrum of solid 4-ATP (**b**); (**D**) The relationship between SERS intensity and 4-ATP concentration at 1077 cm^−1^ and the inset is the linear calibration plot between the SERS intensity and 4-ATP concentration at 1077 cm^−1^ in logarithm scale; (**E**) Raman spectra of 10^−4^ M R6G detected on Au/DW substrate with different periods; (**F**) Plot of Raman intensities of R6G at 1077, 1140 and 1586 cm^−1^ versus different aging time (the error bars were calculated based on 10 independent measurements).

It is common knowledge that sensitivity, stability and reproducibility are essential requirements for any SERS-active substrates. As reported before, the great signal enhancement only occurred when the probe molecules were directly (or closely) adsorbed on the surface of the noble metal [26]. To evaluate the sensitivity of the Au/DW substrate, 4-ATP, which can form a monolayer on the surface of the noble metal due to the existence of mercapto functional group (-SH), was selected as a probe molecule to determine the SERS performance. As shown in Figure 3A, a series of Raman spectra of 4-ATP with different concentrations from 10^−3^ M to 10^−9^ M were obtained by using Au/DW substrate. In addition, the intensity of the Raman signal decreased with the decrease of the concentration. It showed that the characteristic bands at 1586 cm^−1^ of 4-ATP could still be identified even as low as 10^−9^ M (as the shown in Figure 3B which revealed that the LOD of 4-ATP was located at 10^−9^ M for the Au/DW substrate. The strong Raman bands from 4-ATP can be clearly observed at relatively high concentrations and the assignments of the characteristic vibrational modes were given in Table 1 according to Yin et al. [27]. As shown in Figure 3C, relative to the spectrum obtained in the solid, the significant differences in the SERS spectrum Figure 3C(a) on the Au/DW substrate were the frequency shifts for some changes in band intensity. The band shifts from 1087 cm^−1^ in Figure 3C(a) to 1077 cm^−1^ in Figure 3C(a), and the other noticeable Raman frequency shift from 1593 cm^−1^ to 1586 cm^−1^ were also observed. These changes of several representative bands indicated that the -SH group in 4-ATP made direct contact with the Au film surface by forming a strong Au-S bond [28]. Figure 3D shows the relationship between the SERS integrated intensity of the peaks centered at 1077 cm^−1^ and the concentration of 4-ATP, and the inset shows the linear relationship between the Raman intensity and 4-ATP concentrations when both coordinates were under the logarithm function. The error bars in the spectrum were calculated-based upon 10 different measurements. The correlation coefficient was determined to be R^2^ = 0.984 proved the feasibility of Au nanoislands-based bioscaffold substrate for quantitative analysis.

Stability is another important evaluation criterion of a high-performance SERS substrate, therefore, the as-prepared Au/DW substrate functionalized by 10^−4^ M 4-ATP was investigated after long-term storage in the atmosphere. As shown in Figure 3E,F, when the Au/DW substrates treated with different stored time, the Raman intensity got faded down firstly and then tended to be gentle. Choosing the 1586 cm^−1^ peak for example, in the initial detected time of 30 days, the Raman signal intensity of the 4-ATP at 1586 cm^−1^ decreased by 13.20%. After 60 days, the signal of trended to be stable and the SERS intensity fell by 13.32%. Two-month-duration stability tests at other characteristic peaks of 1077 cm^−1^ and 1140 cm^−1^ showed that Raman intensity of 4-ATP reduced only by 9.97% and 14.0%, respectively. The results show that the Au/DW substrate own good SERS performance even after two months of storage, the shape and intensity of peaks keep in stability.

**Figure 4:**
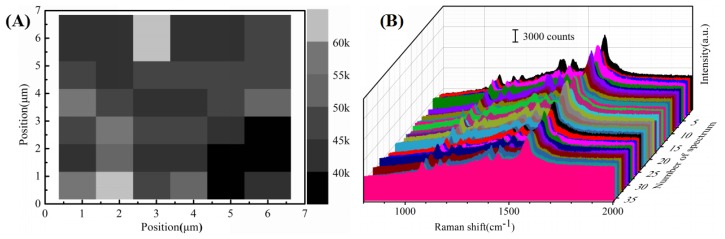
(**A**) SERS mapping (step size is 1 μm) of one same Au/DW substrate. (**B**) Raman spectra of 10^−6^ M 4-ATP obtained from 36 randomly selected spots on Au/DW substrate.

The uniformity and reproducibility of SERS substrates is of crucial importance in quantitative detection because the intensity of the Raman signal depends largely on the homogeneity of the substrate. Therefore, the spot-to-spot uniformity of a Raman mapping on a randomly selected area of 6 μm × 6 μm = 36 μm^2^ for 10^−6^ M 4-ATP was measured across the Au/DW substrate. As shown in Figure 4A, the step size of the mapping was 1 μm and brightness of the grid was proportional to the Raman intensity values based on the integrated area of the baseline-corrected peaks at 1077 cm^−1^. Figure 4B gives the substrate-to-substrate reproducible Raman signals of 10^−6^ M 4-ATP collected from 36 randomly selected spots of 6 substrates. The representative Raman vibrations of 4-ATP molecules at 1077, 1185, 1390 and 1586 cm^−1^ were clearly observed without any shift of characteristic peaks and changes of Raman intensities. To get the statistical results, the relative standard deviation (RSD) values of the Raman signals were calculated according to the following Equation (1) [29]:

(1) $$RSD=\frac{\sqrt{\frac{\sum_{i=1}^{n}{\left(I_{i}-\bar{I}\right)}^{2}}{n-1}}}{\bar{I}},$$

where the $\bar{I}$ stand for the average intensity of the Raman signal, *n* is 36 in agreement with the number of the measured spectrum, $I_{i}$ is the Raman intensity of each spectrum at the same vibrational mode. According to the equation (1), RSD values of the intensities at the major peaks of 4-ATP are shown in Table 2. Obviously, all the RSD values collected from 36 spots were over the range from 9.29% to 12.15%. It has been widely confirmed that a RSD value of the SERS measurement less than 20% over micrometer-sized area indicated high reproducibility of the SERS substrate. The results fully indicated that the Au/DW substrate demonstrated reasonable uniformity and reproducibility across the entire area and there was a great application prospect in pesticide residue detection [30].

**Figure 5:**
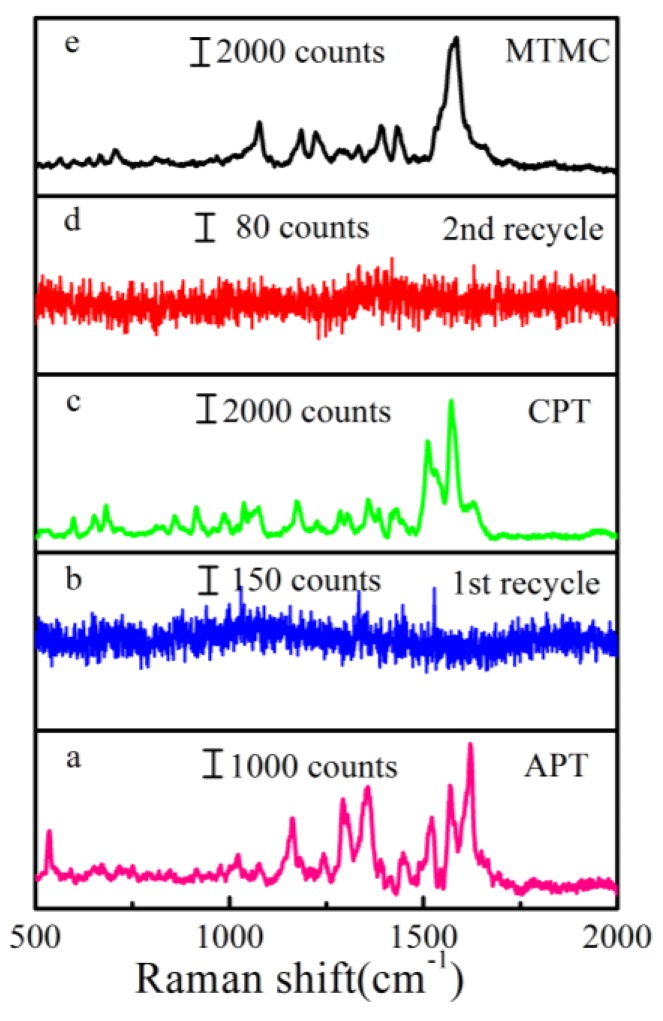
Recyclable SERS behaviors of the alternating analysis of 10^−5^ M APT, CPT and MTMC. Raman spectra of **a**. APT, **b**. CW after first recycle, **c**. CPT, **d**. CW after second recycle and **e**. MTMC

In addition to the sensitivity, stability and reproducibility, recyclability is another important parameter which can effectively reduce the waste of novel metals and the SERS substrates of the biomaterials. Figure 5 shows the results for the three kinds of pesticide collected at initial SERS detection and after washing with NaBH_4_ aqueous solution several times. First, the Au/DW substrate was immersed in to 10^−5^ M APT ethanol solution for 30 min, and then characterized by SERS laser. Subsequently, the APT decorated Au/DW substrate was washed with NaBH_4_ solution for 10 min, then this substrate was washed with deionized water for 4 times. Similarly, the same Au/DW substrate was used to detect MTMC and APT. As shown in Figure 5, the substrate was immersed into the solutions of APT, CPT and MTMC and the SERS signals were obtained. Insets a–e were the Raman signals before and after cleaned by NaBH_4_ aqueous solution, respectively. The phenomenon indicated that the Au/DW substrate had an effective function of recyclability.

### 3.3. Detection of Pesticide Residues Collected by Au/DW Substrate

**Figure 6:**
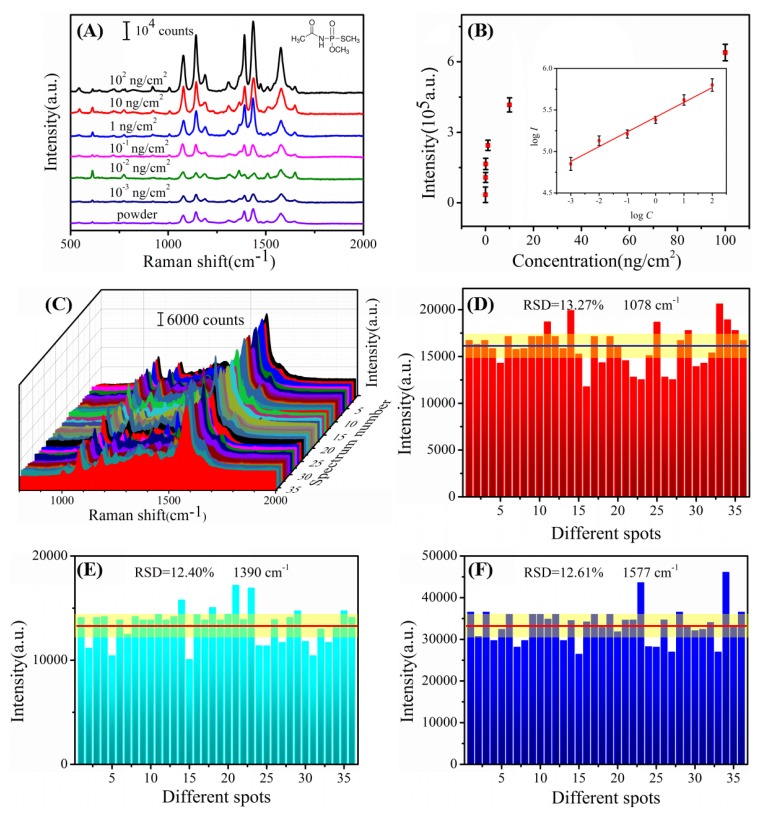
(**A**) SERS spectra of APT with different concentrations of 10^2^–10^−3^ ng/cm^2^ collected from the surface of apple peels and the Raman spectrum of APT powder; (**B**) Raman intensity of APT from apple peels samples at 1577 cm^−1^ by using Au/DW substrate (linear calibration plot between the Raman intensity and acephate concentrations); (**C**) SERS spectra of 10 ng/cm^2^ APT collected from 5 apple peels samples via the “press and peel off” method by using the Au/DW substrate; (**D**–**F**) the main Raman vibrational intensities of 10 ng/cm^2^ APT at characteristic Raman peaks (D: 1078 cm^−1^; E: 1390 cm^−1^; F:1577 cm^−1^).

As previously reported, in the agricultural production of the worldwide, millions of tons of pesticides are annually used to prevent disease and insect pests for the increase of production. However, pesticide residues in agriculture have become an urgent problem that directly threatens human health and the ecological environment [31,32]. For instance, APT, as a kind of organophosphorus pesticides, can effectively defense of chewing type and sucking pests and mites. On the other hand, it may cause poisoning symptoms include dizziness, nausea, vomiting, diarrhea and even coma and respiratory paralysis [33]. Therefore, it is necessary to apply SERS technology to detect pesticide residues in the real world. So far, it has been fully tested that Au/DW substrate exhibited high sensitivity, outstanding stability, and superior reproducibility. Beyond these, with an additional advantage of flexibility, our Au/DW SERS-active substrate was adapted to the detection of APT, CPT and MTMC on apple peels. SERS spectra of the APT with different concentrations from 10^2^–10^−3^ ng/cm^2^ and the Raman spectrum of APT powder were exhibited in Figure 6A. The main Raman bands included 1078, 1145, 1390, 1436 and 1577 cm^−1^ which were the characteristic peaks of APT can be distinguished. A noticeable intensity trend was also observed with concentration. By choosing 1577 cm^−1^ as a representative peak, the LOD was as low as 10^−3^ ng/cm^2^. The LOD and limit of quantitation (LOQ) are shown in Table 3. The LOQ was according to the National food safety standard (GB 2763-2014) in China. The result indicates that the Au/DW substrate satisfied the detection of APT in real application for the LOD was far less than the LOQ. Meanwhile, the intensity at 1577 cm^−1^ peak was further plotted with respect to their concentration, as shown in Figure 6B. The inset shows the linear calibration curve composed by monitoring the Raman intensities centered at 1577 cm^−1^ as a function of concentrations under the scope of logarithmic. Noticeably, log *I* vs. log *C* shows a strong linear relationship where the calculation equation was y = 0.26x + 6.68 and R^2^ was 0.970. The detection of APT molecule proved the feasibility of Au/DW substrate for quantitative analysis.

To further evaluate the reproducibility of Au/DW substrate in particle detection of pesticide residues by this detection method, the SERS spectra of 10 ng/cm^2^ APT which were collected from 4 sets of apple peels by “press and peel off” approach were shown in Figure 6C. The shape of SERS spectra of APT obtained from different positions was very similar. There was neither the shift of the Raman peaks nor the obvious change in the Raman intensity. Meanwhile, the RSD values at three characteristic peaks of 1078, 1390 and 1577 cm^−1^ were calculated to be 13.27%, 12.40% and 12.61% according to Equation (1), respectively, as shown in Figure 6D–F. Uplifting, all the intensities of the characteristic peaks lay within a 14% variation range, revealing the high reproducibility of Au/DW substrate as well as practicality in real detection.

**Figure 7:**
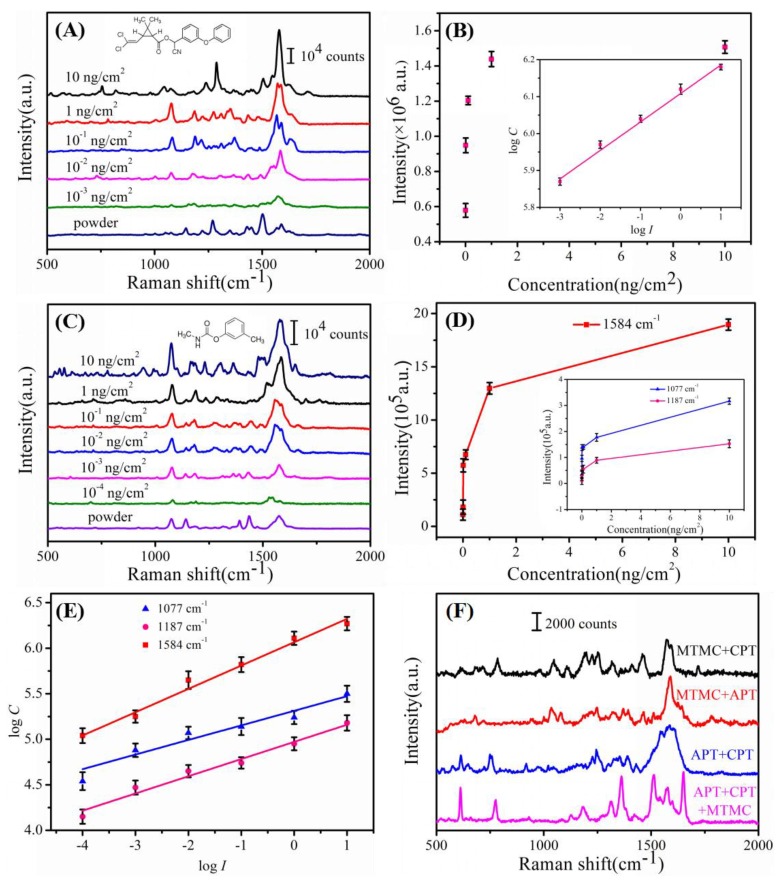
(**A**)The Raman spectra of CPT on the Au/DW substrate from 10 to 10^−3^ ng/cm^2^ and the Raman spectrum of CPT powder. (**B**) the response curve showing the variation of the peak intensities centered at 1578 cm^−1^ as a function of CPT concentrations and the inset shows the response when both axes are converted to a logarithm scale. (**C**)The Raman spectra of MTMC on the Au/DW substrate from 10 to 10^−4^ ng/cm^2^ and the Raman spectrum of MTMC powder. (**D**) Corresponding SERS intensities of main peaks of MTMC at different concentrations. (**E**) Linear calibration plot between the SERS intensity and MTMC concentration. (**F**) SERS spectra of multiple components of pesticide residues (APT, CPT, MTMC) on apple peels using the Au/DW substrate.

Meanwhile, two kinds of pesticides CPT and MTMC which belong to pyrethroid and carbamate pesticides were first detected from apple peels by “press and peel off” approach. SERS spectra of CPT and MTMC were displayed in Figure 7. Figure 7A shows the SERS spectra of Au/DW substrate after being treated by “press and peel off” approach with CPT concentrations ranged from 10–10^−3^ ng/cm^2^, revealing that the SERS signals decreased with the reduction in CPT concentration. It can be clearly seen that the characteristic peaks can be identified down to 10^−3^ ng/cm^2^. Figure 7B shows that the Raman intensities of CPT at 1578 cm^−1^ exhibited an approximately linear growth with the increase in the logarithm of concentration. Meanwhile, as shown in Figure 7C,D, as the concentration of MTMC decreased, the peak intensity gradually decreased. Some scattering peaks of MTMC including 1077 cm^−1^, 1187 cm^−1^ and 1584 cm^−1^ were still distinguishable at concentration of 10^−4^ ng/cm^2^ (Figure 7A). Finally, we observed linear correlations between logarithmic concentration and logarithmic Raman intensity at different characteristic peaks. The calibration curve plotted from the obtained data shows a reliable linear response between the scales of 10 to 10^−4^ ng/cm^2^ as shown in Figure 7E and Table 4. Thus, this Au/DW hybrid can be used as a low cost and highly active SERS substrate for sensing of pesticide residues at apple peels.

### 3.4. Simultaneous Detection of Multifold Pesticide Residues on Apple Peels

In practical production, to prevent the fruits and vegetables from pests and diseases, different pesticides such as organic phosphorus pesticides, carbamate pesticides and pyrethroid pesticides are usually blended and sprayed for farming and for postharvest treatments, which results in multifold pesticide residues on food and thus jeopardizing people’s health [10]. Based on the high performance of the Au/DW substrate analyzed above, this SERS-active substrate can precisely detect and quantify multipesticide residues due to the molecularly narrow band spectra. Therefore, we mixed these three pesticides (APT, CPT and MTMC) and quantified the SERS signal of the mixtures on apple peels by using Au/DW substrate. Figure 7F shows the SERS analysis of mixed pesticides residue. The pesticides mixtures, MTMC + CPT, MTMC + APT, APT + CPT and APT + CPT + MTMC were prepared using equal concentrations (1 ng/cm^2^) of solutions. As shown in Figure 7F, three peaks at 609 cm^−1^, 1391 cm^−1^ and 1577 cm^−1^ were of APT, four peaks at 754 cm^−1^, 1286 cm^−1^, 1505 cm^−1^ and 1578 cm^−1^ were of CPT, and four peaks at 1077 cm^−1^, 1187 cm^−1^, 1516 cm^−1^ and 1584 cm^−1^ were of MTMC. The characteristic peaks of each pesticide residue can be distinctively discerned in the mixed spectrum. The experimental results fully revealed that the proposed method can be used for rapid and on-spot detection of multifold pesticide residues which further demonstrated the powerful analytical ability of the Au/DW substrate in practical applications.

## 4. Conclusions

In summary, a kind of flexible Au/DW substrate was successfully fabricated via a simple and eco-friendly DC magnetron sputtering system. Raman measurements showed that the fabricated 3D biomimetic arrays can indeed as high-performance SERS-active substrate. Sensitivity tests revealed that the synthesized Au/DW substrate showed high SERS sensitivity for 4-ATP and the LOD of 1 × 10^−9^ M was obtained which meet the requirements of ultra-sensitive detection. Two months of continuous stability research showed Raman intensity of 4-ATP reduced only by 14.0% after aging for two mouths. The maximum value of RSD was less than 12.15% from different positions on the same substrate, revealing outstanding uniformity and reproducibility. More importantly, multifold pesticide residues detections were demonstrated when the Au/DW substrate was used in detecting APT, CPT and MTMC at apple peels via a simple “press and peel off” method, with the LODs of 10^−3^ ng/cm^2^, 10^−3^ ng/cm^2^ and 10^−4^ ng/cm^2^, respectively. Meanwhile, multicomponent pesticide residues detection indicated that the high-performance and flexible SERS substrate can be employed for rapid sampling from arbitrary curved surfaces and multicomponent analysis in real samples. Therefore, the flexible Au-decorated DW bioscaffold arrays can be a potential candidate in food safety and environmental monitoring.

## Figures and Tables

**Table 1:** Experimental assignments of vibrations of 4-ATP solid and 4-ATP on the Au/DW substrate (*ν*, stretching; *δ*, bending; *γ*, out-of-plane deformation (respect to the benzene ring)).

Modes	Solid	SERS	Band Assignment
11(*b_1_*)	825		*π_CH_*
18a(*a_1_*)	1005	1007	*γ_CC_ + γ_CCC_*
7a(*a_1_*)	1087	1077	*ν_CS_*
9b(*b_2_*)		1140	*δ_CH_*
9a(*a_1_*)	1177	1185	*δ_CH_*
7a(*a_1_*)	1290		*ν_CH_*
3b(*b_2_*)		1390	
19a(*a_1_*)	1492		*ν_CC_ + δ_CH_*
19b(*b_2_*)		1443	*ν_CC_ + δ_CH_*
8a(*a_1_*)	1593	1586	*ν_CC_*

**Table 2:** RSD values for the major peaks of the 4-ATP SERS spectrum.

Raman peaks (cm^−1^)	1077	1140	1185	1390	1443	1586
RSD values	9.29%	9.95%	9.85%	10.60%	10.36%	12.15%

**Table 3:** Analytical figures of merit for the quantitative SERS detection of APT residues collected from apple peels (The concentration converted from mass-to-area ratio to mg per kilogram, was roughly calculated according to previous work [32]).

Fruit Peels	LOD	LOQ
Apple	1 × 10^−3^ ng/cm^2^	0.625 ng/cm^2^

**Table 4:** Linear relationships between MTMC concentrations (10 ng/cm^2^–10^−4^ ng/cm^2^) and Raman intensities at characteristic peaks of MTMC.

Peak/cm^−1^	Linear Function	R^2^
1077	y = 0.16x + 5.31	0.911
1187	y = 0.19x + 4.97	0.967
1584	y = 0.26x + 6.07	0.985
